# Chronic subdural hematoma management in Thailand: a nationwide survey of clinical practice and trends

**DOI:** 10.3389/fneur.2025.1636167

**Published:** 2025-09-01

**Authors:** Todsapon Praphanuwat, Chanon Srihagulang, Kriengsak Limpastan, Thunya Norasethada, Tanat Vaniyapong, Wanarak Watcharasaksilp, Chumpon Jetjumnong, Phonphimon Ruankham, Jirapong Vongsfak

**Affiliations:** ^1^Division of Neurosurgery, Department of Surgery, Faculty of Medicine, Chiang Mai University, Chiang Mai, Thailand; ^2^Clinical Surgical Research Center, Department of Surgery, Faculty of Medicine, Chiang Mai University, Chiang Mai, Thailand; ^3^Division of Geriatric Medicine, Department of Medicine, Faculty of Medicine, Chiang Mai University, Chiang Mai, Thailand; ^4^Department of Radiology, Faculty of Medicine, Chiang Mai University, Chiang Mai, Thailand

**Keywords:** chronic subdural hematoma, survey study, nationwide, burr hole drainage, MMA embolization

## Abstract

**Background:**

Chronic subdural hematoma (CSDH) presents a growing challenge in neurosurgical care due to its increasing incidence, recurrence rates, and aging-related risk factors. Treatment approaches vary widely, particularly in Southeast Asia, where limited data exists regarding standardized management. This study aims to evaluate current clinical practices and emerging treatment trends for CSDH among Thai neurosurgeons.

**Methods:**

A nationwide cross-sectional survey was conducted in January 2025 among practicing neurosurgeons across Thailand. The 29-item web-based questionnaire covered demographics, surgical techniques, adjunct therapies, and postoperative care. Descriptive statistics were used to summarize responses. Results were synthesized and compared with previously published nationwide surveys to identify practice trends and deviations.

**Results:**

A total of 53 responses were received (76% response rate). Most respondents practiced in public hospitals (71.7%) and managed 10–30 CSDH cases annually (56.6%). The preferred surgical method was two burr-hole craniostomy (73.6%), with 96.2% of surgeons using intraoperative irrigation, predominantly with room-temperature normal saline. Drain placement was nearly universal (96.2%), with 72.5% opting for subdural drains. Postoperative care varied, with 30.2% prescribing 48-h bed rest and 66% not performing routine postoperative CT scans. Corticosteroids were used by 37.7%, mostly in conservative cases, while 40% prescribed statins, and 39.6% utilized tranexamic acid. Adjunctive middle meningeal artery (MMA) embolization was employed by 45.3% of respondents. Decisions to resume antiplatelet or anticoagulant therapy were guided by postoperative imaging in over half of the cases.

**Conclusion:**

This survey highlights substantial heterogeneity in the management of chronic subdural hematoma (CSDH) across Thailand. While burr-hole craniostomy with irrigation and drainage remains the standard surgical approach, notable variations exist in medical therapy and postoperative care. The widespread use of middle meningeal artery (MMA) embolization and other adjunctive therapies reflects evolving treatment trends. These findings emphasize the need for national, evidence-based guidelines to standardize care, reduce variability, and improve patient outcomes.

## Introduction

Chronic subdural hematoma (CSDH) is a neurological condition involving the accumulation of blood and cerebrospinal fluid in the subdural space. It poses significant challenges in neurosurgery due to high incidence and recurrence rates, ranging from 2.6 to 33% depending on treatment modalities (1, 2). CSDH typically develops several weeks after minor head trauma. Its prevalence is expected to rise globally with the aging population and increased use of anticoagulant and antiplatelet medications (1, 3, 4).

Treatment decisions for CSDH primarily depend on neurological symptoms and evidence of brain compression. Asymptomatic cases without compression are generally managed conservatively or medically. Medical options include corticosteroids, tranexamic acid (TXA), statins, and ACE inhibitors (5–7). Recently, middle meningeal artery (MMA) embolization has emerged as an alternative treatment option, either as a primary therapy or an adjunct to surgical treatment, for chronic subdural hematoma (CSDH), demonstrating effectiveness and a low complication rate (8). Despite various medications being used, evidence is limited, leading to varied approaches and a focus on individualized treatment strategies.

Surgical options for CSDH include twist-drill craniostomy, burr-hole craniostomy, and mini-craniotomy. The choice depends on factors like hematoma configuration and age. Although burr-hole craniostomy is often seen as more effective, it is not always preferred due to these considerations. No universally accepted first-line surgical treatment exists, with techniques like single or double burr-hole craniostomy in use. The optimal surgical technique remains debated.

The key in managing symptomatic CSDH patients is hematoma evacuation. Surgical techniques, adjuvant therapies, and peri-operative management vary across centers. Previous surveys have shown significant heterogeneity in CSDH treatment, but few studies focus on Asia, particularly Southeast Asia. This study aims to investigate variations in CSDH management across neurosurgical units in Thailand to support the development of standardized treatment protocols.

## Materials and methods

### Study design and ethics approval

In January 2025, a web-based survey was developed using Google Forms. The questionnaire included 29 items addressing demographic information, surgical techniques, and perioperative and postoperative care for patients with chronic subdural hematoma (Table 1). Prior to data collection, ethics exemption was obtained from the Research and Ethics Committee (STUDY CODE: SUR-2568-0098).

**Table 1:** Distributed online survey.

Question	Answers
Demographics data	
1. Where is the location of your practicing hospital?	Northern region/ Northeastern region/Central region/Western region/ Southern region/Bangkok/Eastern region
2. What is type of your hospital?	Public hospital/ Private hospital/University Hospital
3. How many cases of chronic subdural hematoma have you previously managed? (per year)	<10 cases/10–30 cases/>30 cases
Surgical technique	
4. Which surgical techniques are your preferred choice for initial treatment of unilateral symptomatic uncomplicated chronic subdural hematoma?	Twist-drill craniostomy/Single burr hole/Double burr hole/Craniotomy/Minicraniotomy irrigation
5. What surgical methods are used to remove a hematoma from the subdural space?	Only use fluid irrigation in subdural space until Clear/Only remove outer membrane/Open and remove outer and inner membrane
Fluid irrigation	
6. Do you utilize intraoperative fluid irrigation during surgery?	Yes/No
7. If yes to question 6, what type of fluid irrigation do you typically use?	Ringer lactate solution/Normal saline solution
8. If yes to question 6, What temperatures are used for fluid irrigation during the management of chronic subdural hematoma?	Room temperature/Body temperature
Drain use	
9. Do you use a retained drain?	Yes/No
10. If yes to question 9, Where is the drain usually positioned in cases of chronic subdural hematoma?	Subgaleal space/Ubdural space
11. Which burr hole is preferred for placing the drain?	Anterior hole/Posterior hole
12. What types of drains are commonly used?	Non-suction drain/Low pressure suction drain
13. When is the drain typically removed after surgery?	< 24 h/ < 48 h/ < 72 h/Drain stops draining
14. If you answer B.subdural space to question 10, How long is the drain typically left in the subdural space?	Just placed in hole/1–2 cm/ > 2 cm
Postoperative management/Adjunct therapy	
15. Do you routinely request a postoperative CT scan?	Immediate postoperative/Before remove drain/Before discharge/No routine postoperative CT scan
16. What is the duration of postoperative bed rest?	24 h/ 48 h/ > 48 h/No bedrest
17. Do you prescribe corticosteroids for patients with chronic subdural hematoma?	Every case/Only conservative cases/Only surgical cases/Never used
18. If a corticosteroid has been used previously, what preparation would you prescribe for the use of corticosteroids?	Intravenous dexamethasone/Oral dexamethasone/Oral prednisolone
19. If a corticosteroid has been used previously, What is the recommended duration for which you prescribe corticosteroids?	< 4 weeks/ 4–8 weeks/Until hematoma resolution
20. Do you prescribe statin for patients with chronic subdural hematoma?	Every case/ Only conservative cases/Only surgical cases/Never used
21. What is the typical daily dosage of statins that you would prescribe?	10 mg/ 20 mg/ 40 mg
22. How long would you typically prescribe a statin?	< 4 weeks/ 4–8 weeks/ > 8 weeks/ Until hematoma resolution
23. Do you prescribe tranexamic acid for patients with chronic subdural hematoma?	Every case/Only conservative cases/Only surgical cases/Never used
24. What is your recommended duration for postoperative antibiotic use?	24 h/72 h/5 days/Until drain removal
25. What is the recommended time for scheduling a follow-up CT scan at the outpatient clinic?	1 week/2 weeks/4 weeks/3 months/Not routine
26. After the removal of a chronic subdural hematoma, when is it appropriate to resume aspirin therapy postoperatively?	Depend on CT scan/ < 2 weeks/ 2–4 weeks/Depend on indication of aspirin prescription
27. After the removal of a chronic subdural hematoma, when is it appropriate to resume anticoagulant therapy postoperatively?	Depend on CT scan/ < 2 weeks/ 2–4 weeks/Depend on indication of aspirin prescription
28. Do you utilize adjunctive MMA embolization in your treatment?	Yes/No

### Survey distribution and participants

The survey link was distributed exclusively through the Thai Neurosurgical Society and affiliated professional networks, thereby restricting access to the intended target population. Respondents were instructed that only board-certified neurosurgeons should participate. To enhance response verification, professional identification such as hospital affiliation, clinical role, and estimated case volume was collected.

### Response management and confidentiality

Two weeks after the initial invitation, reminder emails were sent to all potential participants. Respondents were assured of complete data confidentiality throughout the process.

### Data analysis

Responses were recorded anonymously in a Microsoft Excel database. Descriptive data were presented as proportions using STATA version 14. Differences in response rates were evaluated using the Chi-square test. A *p*-value of < 0.05 was considered statistically significant.

### Results

Of the 70 surveys that were received at the recipient e-mail address, 53 were returned and thus response rate was 76%. Therefore, this study is based on the analysis of 53 neurosurgeons working different part of country.

### Demographic

Most respondents (34%) were from the northern region of Thailand, followed by Bangkok (24.5%), the central region (17%), the northeastern region (11.3%), and the southern region (11.3%). The primary type of hospital attended was public hospitals (71.7%), followed by university hospitals (15.1%) and private hospitals (13.2%). The number of chronic subdural hematoma cases treated annually was predominantly between 10 and 30 cases (56.6%), with 24.5% of respondents treating more than 30 cases per year (Table 2).

**Table 2:** Demographics of responders.

%Variable	*n* (%)
Location of practiced hospital	
Northern region	18 (34)
Northeastern region	6 (11.3)
Central region	9 (17)
Western region	0
Southern region	6 (11.3)
Bangkok	13 (24.5)
Eastern region	1 (1.9)
Type of hospital	
Public hospital	38 (71.7)
Private hospital	7 (13.2)
University hospital	8 (15.1)
No. of cSDH per year	
< 10	10 (18.9)
10–30	30 (56.6)
> 30	13 (24.5)

### Surgical treatment and intraoperative technique

The most common surgical technique for symptomatic chronic subdural hematoma (CSDH) was two burr-hole craniostomy (73.6%), followed by craniotomy (17%), single burr-hole craniostomy (5.7%), and minicraniotomy (3.8%). Subdural hematoma evacuation was primarily performed using fluid irrigation until the effluent was clear (69.8%). In cases managed with craniotomy, resection of both the outer and inner membranes was performed in 22.6% of procedures (Table 3).

**Table 3:** Response to type of treatment.

Variable	*n* (%)
Surgical technique for symptomatic CSDH	
Twist-drill craniostomy	0
Two burr hole	39 (73.6)
Single burr hole	3 (5.7)
Craniotomy	9 (17)
Minicraniotomy irrigation	2 (3.8)
Technique subdural removal	
Only use fluid irrigation in subdural space until clear	37 (69.8)
Only remove outer membrane	4 (7.6)
Open and remove outer and inner membrane	12 (22.6)
Use intraoperative fluid irrigation	
Yes	51 (96.2)
No	2 (3.8)
Type of fluid irrigation	
Normal saline solution	51 (100)
Ringer lactate solution	0
Temperature of fluid irrigation	
Room temperature	38 (74.5)
Body temperature	13 (25.5)
Drain insertion	
Yes	51 (96.2)
No	2 (3.8)
Drain location	
Subdural space	37 (72.5)
Subgaleal space	14 (27.5)
Specific drain location	
Anterior hole	27 (52.9)
Posterior hole	24 (47.1)
Type of drain	
Non-suction drain	37 (72.5)
Low pressure suction drain	14 (27.5)
Duration of drain placement	
< 24 h	0
< 48 h	8 (15.7)
< 72 h	30 (58.8)
Drain stops draining	13 (25.5)
Length of drain placement	
Just placed in hole	17 (45.9)
1–2 cm	13 (35.1)
> 2 cm	7 (18.9)
Postoperative CT scan	
Immediate postoperative	4 (7.5)
Before remove drain	8 (15.1)
Before discharge	6 (11.3)
No routine postoperative CT scan	35 (66)
Bedrest duration	
24 h	13 (24.5)
48 h	16 (30.2)
> 48 h	10 (18.9)
No bedrest	14 (26.4)
Corticosteroid used	
Every case	3 (5.7)
Only conservative cases	15 (28.3)
Only surgical cases	2 (3.8)
Never used	33 (62.3)
Type of corticosteroid prescription	
Intravenous dexamethasone	9 (45)
Oral dexamethasone	4 (20)
Oral prednisolone	7 (35)
Duration of corticosteroid	
< 4 weeks	14 (70)
4–8 weeks	4 (20)
Until hematoma resolution	2 (10)
Statin used	
Every case	11 (20.8)
Only conservative cases	8 (15.1)
Only surgical cases	2 (3.8)
Never used	32 (60.4)
Dose of statin	
10 mg	2 (9.5)
20 mg	8 (38.1)
40 mg	11 (52.4)
Duration of statin	
< 4 weeks	4 (19)
4–8 weeks	8 (38.1)
> 8 weeks	3 (14.3)
Until hematoma resolution	6 (28.6)
Tranexamic acid used	
Every case	11 (20.8)
Only conservative cases	1 (1.9)
Only surgical cases	9 (17)
Never used	32 (60.4)
Duration of postoperative antibiotic	
24 h	26 (49.1)
72 h	18 (34)
5 days	2 (3.8)
Until drain removal	7 (13.3)
CT Scan during OPD visit	
1 week	2 (3.8)
2 weeks	18 (34)
4 weeks	27 (51)
3 months	1 (1.9)
Not routine	5 (9.5)
Resume aspirin postoperative	
Depend on CT scan	33 (62.3)
< 2 weeks	6 (11.4)
2–4 weeks	12 (22.6)
Depend on indication of aspirin prescription	2 (3.8)
Resume anticoagulant postoperative	
Depend on CT scan	31 (58.5)
< 2 weeks	6 (11.3)
2–4 weeks	14 (26.4)
Depend on indication of anticoagulant prescription	2 (3.8)
Adjunctive MMA embolization	
Yes	24 (45.3)
No	29 (54.7)

Intraoperative fluid irrigation with normal saline was used in 96% of cases. Among these, room temperature saline was preferred in 74.5% of procedures, while body temperature saline was used in 25.5%.

Postoperative drain insertion was performed in 96.2% of patients. Among these, subdural drains were placed in 72.5% and subgaleal drains in 27.5%. The drain was most commonly placed through the anterior burr hole (52.9%) compared to the posterior hole (47.1%).

Regarding the type of drain, non-suction drains were used in 72.5% of cases, whereas low-pressure suction drains were used in 27.5%. The depth of drain placement varied: 45.9% were placed just within the burr hole, 35.2% were inserted 1–2 cm, and 18.9% extended beyond 2 cm into the subdural space.

### Post operative care

Postoperative bed rest was commonly prescribed, with 30.2% of patients advised to rest for 48 h, followed by 24.5% for 24 h, and 18.9% for more than 48 h. Conversely, 26.4% of patients were not required to undergo bed rest after surgery.

The duration of drain placement was typically less than 72 h in 58.8% of cases. In 25.5%, the drain was left in place until drainage ceased, while in 15.7%, the drain was removed early, within 48 h postoperatively.

Routine postoperative CT brain imaging was not performed in the majority of cases (66%). A CT scan prior to drain removal was obtained in 15.1%, and a CT scan before discharge was performed in 11.3% of patients.

Regarding antibiotic prophylaxis, 49% of cases received antibiotics within 24 h, 34% continued for up to 72 h, and 13% received antibiotics until the drain was removed.

### Medical treatment and follow-up imaging

The most commonly used medication for CSDH treatment was corticosteroids, with 45% of patients receiving intravenous dexamethasone, followed by 35% treated with oral prednisolone, and 20% with oral dexamethasone. The duration of corticosteroid therapy was typically less than 4 weeks in 70% of cases, 4 to 8 weeks in 20%, and until hematoma resolution in 10%.

Regarding statin therapy, 60% of patients did not receive statins, while 20.8% were treated with statins as part of standard care, and 15% received statins only in conservatively managed cases. The most commonly prescribed dose was 40 mg per day, followed by 20 mg per day. The duration of statin treatment was 4–8 weeks in 38% of cases and continued until hematoma resolution in 28.6%.

Tranexamic acid was used in 21% of cases, with 17% receiving it exclusively in surgically treated patients. However, 60.4% reported no use of this medication in CSDH management.

For follow-up imaging, a CT brain scan during outpatient clinic (OPD) follow-up was most commonly scheduled at 4 weeks post-discharge (50%), followed by 2 weeks (34%). 10% of patients did not undergo routine follow-up CT imaging.

### Middle meningeal artery (MMA) embolization

Adjunctive middle meningeal artery (MMA) embolization was performed in 45.3% of patients as part of the treatment strategy for CSDH.

### Management of patients on antiplatelet or anticoagulant therapy

The timing of resuming antiplatelet (aspirin) and anticoagulant medications postoperatively was primarily guided by follow-up CT brain imaging in 62.3 and 58.5% of cases, respectively. In other cases, therapy was resumed after 2–4 weeks in 22% (aspirin) and 26.4% (anticoagulants). Approximately 11% of patients had both antiplatelet and anticoagulant therapy resumed within 2 weeks post-surgery.

## Discussion

This nationwide survey provides a comprehensive overview of current practices in the management of chronic subdural hematoma (CSDH) among neurosurgeons in Thailand. The majority of respondents favored two burr-hole craniostomy as the primary surgical technique, with fluid irrigation and drain placement being nearly universal intraoperative steps. Postoperative care varied, particularly regarding bed rest duration, drain management, and imaging follow-up. The study also highlights evolving trends in medical therapy, such as the use of corticosteroids and statins, and the increasing adoption of middle meningeal artery (MMA) embolization as an adjunctive treatment. Importantly, there was significant heterogeneity in the resumption of antiplatelet and anticoagulant therapy, often guided by postoperative imaging. These findings reflect both adherence to common practices and variability that may benefit from future standardization. To contextualize our findings, we compared them with previous nationwide surveys conducted in other regions. A detailed summary is presented in Table 4.

**Table 4:** Comprehensive international comparison of management practices in chronic subdural hematoma (CSDH).

Management aspect	Cenic et al. (22)	Santarius et al. (12)	Javadi et al. (23)	Rabiu et al. (11)	Avanali et al. (24)	Soleman et al. (10)	Teles and Kraemer (25)	Baschera et al. (26)	Holl et al. (27)	Praphanuwat et al. (28)
Participants	Canadian neurosurgeons	Neurosurgeons in UK and Ireland	Neurosurgeons in Iran	Neurosurgeons in Nigeria	Neurosurgeons in India	Global neurosurgeons (mainly in Europe)	Brazilian neurosurgical departments	Neurosurgeons in Germany, Austria and Switzerland	Dutch neurologists and neurosurgeons	Thai neurosurgeons
Preferred surgical technique	Two burr-holes (49.5%)	Two burr-holes (92%)	Two burr-holes (64%)	BH 50% one, 50% two	Two burr-holes (59%)	Two burr-holes (56%)	One BH 42.9%, Two BH 42.9%	One burr-hole (65%)	One burr-hole (68.1%)	Two burr-holes (73.6%)
Techniques subdural removal	Not mentioned	Not mentioned	Not mentioned	Not mentioned	Irrigation to subdural space until clear (82%)	Not mentioned	Not mentioned	Use drain (Nelaton) to rinse in the area further from access point (52%)	Use fluid flush in subdural space (95.9%)	Only use fluid irrigation in subdural space until clear (69.8%)
Intraoperative irrigation	Not mentioned	Not mentioned	Not mentioned	Saline with antibiotics (78%)	Not mentioned	Not mentioned	Not mentioned	Isotonic crystalloid solution	Body temperature fluid (78.7%) and physiological salt solution (58.7%)	Room temp saline (74.5%)
Drain use (%)	80.6% (Jackson-Pratt, subdural)	Not routine used (mainly use <25% of cases)	Common	42.9% (mainly non-suction, subdural)	29.5% routine used, 42.6% selectively placed	80% (mainly subdural)	83.7% (mainly subdural)	~90% (non-suction, subdural)	97.9% (mainly subdural)	96.2% (mainly non-suction, subdural)
Length of drain placement	Not mentioned	Not mentioned	Not mentioned	Not mentioned	Not mentioned	Not mentioned	Not mentioned	Not mentioned	Not mentioned	Just within burr hole (45.9%)
Duration of drain placement	Not mentioned	Not mentioned	Not mentioned	48 h (50%)	Majority remove within 2 days	48 h (76%)	48 h (56.1%)	When it stops draining (26%), after 48 h (26%)	24–48 h (63.8%)	Within 72 h (58.8%)
Bedrest duration	1 day (52.5%)	1–2 days (57%)	Not mentioned	2 days (50%)	Not mentioned	Not mentioned	< 2 days (75.5%)	Not bedrest (66%)	Bedrest as long as drainage system is connected (66.7%)	48 h (30.2%)
Post-op antibiotics duration	Pre-operative only (70.1%)	Not mentioned	Not mentioned	Not mentioned	Not mentioned	Single dose (58%)	Not mentioned	Only single dose before incision (87%)	Not mentioned	Within 24 h (49%)
Routine postop CT imaging	Not mentioned	Not routine (68%)	Not mentioned	Not routine (78%)	Routine at 3 months (59%)	Routine (59%)	Routine (65.3%)	Routine (87%)	Routine (65%)	Not routine (66%)
Medical treatment	CS (13.3%)	CS 55% in conservative	Not mentioned	Not use	CS (21% in selected patients)	CS (rare, 2%)	CS (2%)	CS (23%), TXA (45%)	CS (selective), TXA/statins (rare)	CS (37.7%), TXA (39.6%), statins (39.6%)
Adjunctive MMA embolization	Not mentioned	Not mentioned	Not mentioned	Not mentioned	Not mentioned	Not mentioned	Not mentioned	Not mentioned	MMA embolization (4.3%)	MMA embolization (45.3%)
Resume antiplatelet/anticoagulant	Not mentioned	Not mentioned	Not mentioned	Not mentioned	based on indication and follow up CT	Not mentioned	Not mentioned	Based on follow-up CT	Not mentioned	Based on follow-up CT (~60%)

Surgical intervention remains the gold standard for managing symptomatic chronic subdural hematoma (CSDH), particularly in cases with significant mass effect, such as a hematoma thickness >10 mm or midline shift >5 mm. In alignment with current international practice, our study found that two burr-hole craniostomy was the most commonly performed technique (73.6%) among Thai neurosurgeons. This preference is supported by previous literature demonstrating a relatively low recurrence rate of approximately 12% and a morbidity rate of 4%, compared to higher rates associated with twist-drill craniostomy (9). The use of two burr holes provides better exposure and access for fluid evacuation compared to a single burr hole, which is typically reserved for small, localized hematomas or for patients undergoing surgery under local anesthesia. In our cohort, 72.5% of respondents placed the drain in the subdural space, with the majority (58.8%) removing it within 72 h postoperatively. Current literature suggests no significant difference in recurrence rates between subdural and subgaleal/subperiosteal drain placement, indicating that both locations may be viable depending on surgeon preference (9). Similarly, the choice between frontal versus parietal burr hole placement for drain insertion does not appear to influence recurrence rates in a statistically significant manner.

In our study, 49% of respondents reported prescribing antibiotic prophylaxis for 24 h, which contrasts with findings from a survey by Soleman et al., where 84% of respondents administered a single prophylactic dose prior to skin incision (10). This discrepancy reflects the ongoing variation in infection prevention strategies in CSDH surgery and highlights the need for consensus guidelines to balance efficacy with antibiotic stewardship.

Regarding postoperative imaging, 34% of respondents in our survey routinely obtained a postoperative CT scan, a rate that falls within the range reported in previous studies (21–68%) (10–12). This variation suggests a lack of standardized imaging protocols post-CSDH evacuation, with decisions often guided by individual clinical judgment or institutional policy rather than uniform criteria.

Corticosteroids have been investigated as a potential adjunctive therapy for CSDH due to their anti-inflammatory effects, including the inhibition of vascular endothelial growth factor (VEGF) production, which plays a role in hematoma membrane formation and recurrence (13). While several case series have reported a reduction in recurrence rates, the evidence remains limited. In our study, 62% of respondents reported not using corticosteroids, a finding consistent with results from large randomized controlled trials such as the Dex-CSDH trial and the DECSA study conducted in the UK and the Netherlands (5, 14). These trials failed to demonstrate significant clinical benefit of corticosteroids over placebo when used in conjunction with surgery and were associated with an increased incidence of serious adverse events, including infections.

An alternative pharmacological agent currently gaining traction is atorvastatin, which possesses both cholesterol-lowering and anti-inflammatory properties, and is thought to promote endothelial progenitor cell mobilization, proliferation, and migration (15). In our study, 40% of respondents prescribed statins, most commonly at a dosage of 40 mg per day for a duration of 4–8 weeks. This practice is supported by recent meta-analyses, which suggest that statins may help reduce hematoma recurrence and volume, particularly in conservatively managed patients, without significant adverse effects (16). However, the clinical utility of statins still requires validation through larger, high-quality randomized trials.

Tranexamic acid, an antifibrinolytic agent that also modulates inflammation, was used in 40% of respondents’ practices. Emerging evidence suggests its efficacy in reducing recurrence rates without increasing the risk of myocardial infarction or thromboembolic events, making it a potentially valuable adjunct in selected patients (17).

Middle meningeal artery (MMA) embolization has emerged over the past decade as a safe and effective alternative or adjunct to surgical treatment for chronic subdural hematoma (CSDH). In our study, 45.3% of respondents reported utilizing adjunctive MMA embolization as part of their treatment approach. Interestingly, Thailand had one of the highest reported rates of adjunctive middle meningeal artery (MMA) embolization (45.3%), significantly higher than prior studies where its use was rare or not reported at all. This suggests a rapid adoption of this technique in Thailand compared to international counterparts. Similarly, medical therapies such as corticosteroids, tranexamic acid, and statins were more commonly reported among Thai respondents than in earlier surveys from Europe or South America, reflecting emerging evidence and potentially more proactive adjunctive treatment strategies. The therapeutic rationale for MMA embolization is to interrupt the pathological cycle of recurrent bleeding by occluding the dural vessels that supply the neomembranes responsible for chronic inflammation (18). This process not only prevents further hemorrhage but may also facilitate gradual resorption of subdural fluid. Recent systematic reviews and meta-analyses, including studies published in 2023, have demonstrated that MMA embolization significantly reduces treatment failure rates and the need for rescue surgical intervention, while also promoting complete hematoma resolution in both primary and recurrent cases (19). These findings support the growing integration of MMA embolization into the standard management algorithm, particularly for patients at high surgical risk or those with recurrent disease.

The use of aspirin and anticoagulants is known to increase the risk of bleeding and recurrence in patients with chronic subdural hematoma (CSDH). A systematic review published in 2017 demonstrated that anticoagulant use was significantly associated with an increased recurrence rate of subdural hematoma (20). Consequently, the timing of resuming these medications postoperatively must be carefully considered.

In our study, 62.3 and 58.5% of respondents reported that the decision to resume antiplatelet and anticoagulant therapy, respectively, was based on postoperative CT brain imaging to ensure patient safety. These findings are consistent with prior surveys, including a literature review in which 62% of clinicians performed imaging prior to resuming antiplatelet therapy (21). This practice reflects a cautious, imaging-guided approach aimed at minimizing the risk of rebleeding while balancing thromboembolic risk.

The variability in perioperative care, surgical techniques, and postoperative management highlights the need for standardized national guidelines for the treatment of chronic subdural hematoma (CSDH) in Thailand. These data can provide a foundation to support guideline development, which could help reduce practice variation, improve patient outcomes, and promote efficient resource utilization.

Building on these findings, future research should focus on generating outcome-based evidence to inform clinical practice and policy. Prospective studies are needed to evaluate the efficacy, safety, and cost-effectiveness of adjunctive therapies, such as MMA embolization, statins, corticosteroids, and tranexamic acid, within the Thai healthcare context. In addition, randomized controlled trials comparing surgical techniques, drain placement strategies, and postoperative protocols would be instrumental in establishing standardized care pathways. Implementation science research is also warranted to identify barriers and facilitators to the adoption of evidence-based guidelines across diverse hospital settings nationwide.

This study has some limitations. The results are based on self-reported data from a survey, which may be affected by recall bias or personal interpretation. The sample size was relatively small, with 53 neurosurgeons, and may not fully reflect nationwide practices. Also, we did not assess patient outcomes, so we cannot link specific treatments to success or recurrence rates. Lastly, differences in hospital resources or protocols were not explored, which may influence individual practices. Despite these limitations, the study provides useful insights into current CSDH management trends in Thailand.

## Conclusion

This nationwide survey highlights current practices in the management of chronic subdural hematoma (CSDH) among neurosurgeons in Thailand. Burr-hole craniostomy with closed-system drainage remains the standard and most widely adopted surgical approach, reflecting global best practices. Although corticosteroids, such as dexamethasone, have been explored for their anti-inflammatory properties, recent high-quality randomized trials and our findings suggest no significant benefit in treatment success or mortality, with a concerning increase in adverse effects.

In contrast, atorvastatin and tranexamic acid are gaining traction as promising adjunctive therapies, with emerging evidence supporting their role in reducing recurrence rates without major safety concerns. Middle meningeal artery (MMA) embolization has also emerged as a valuable adjunct or alternative, particularly for recurrent or high-risk cases.

Overall, our findings underscore the importance of aligning clinical practice with evolving evidence and highlight areas where future consensus guidelines and high-quality research are needed to optimize outcomes in CSDH management. By identifying real-world practice patterns and treatment variability, this study enhances the understanding of CSDH care in Thailand and reveals opportunities to support standardization and drive evidence-based improvements in clinical practice.

## Data Availability

The datasets presented in this study can be found in online repositories. The names of the repository/repositories and accession number(s) can be found in the article/supplementary material.

## In memoriam

We would like to express our deepest gratitude to Associate Professor Kriengsak Limpastan, our great mentor, beloved teacher, and former Head of the Neurosurgery Unit, Faculty of Medicine, Chiang Mai University, as well as former President of the Royal College of Neurological Surgeons of Thailand. His vision, leadership, and dedication laid the foundation for our academic and clinical excellence. Professor Kriengsak was a true pillar of our neurosurgical community whose sudden passing in May 2025 is a profound loss to all of us. This study is dedicated in his loving memory, with heartfelt appreciation for the inspiration, wisdom, and guidance he generously shared throughout his life.
